# Multiplex PCR for Microbiological Testing in Patients with Peritoneal Dialysis- and Liver Cirrhosis-Related Peritonitis: Faster, but Less Sensitive

**DOI:** 10.3390/jcm14082641

**Published:** 2025-04-11

**Authors:** Sebastian Schwab, Daniel Pörner, Dominik Boes, Achim Hoerauf, Jacob Nattermann, Christian Strassburg, Gunnar T. R. Hischebeth, Philipp Lutz

**Affiliations:** 1 Department of Internal Medicine I, University of Bonn, 53127 Bonn, Germany; 2 Kuratorium for Dialysis, KfH Renal Center, 53127 Bonn, Germany; 3 Institute of Medical Microbiology, Immunology and Parasitology, University Hospital of Bonn, 53127 Bonn, Germany

**Keywords:** peritonitis, liver cirrhosis, peritoneal dialysis, PCR

## Abstract

**Background:** We analyzed the performance of a multiplex PCR application (Unyvero IAI) in comparison to culture in a cohort of peritonitis patients undergoing peritoneal dialysis or with liver cirrhosis. **Methods:** We performed a single-center study of 47 patients with clinically suspected peritonitis and compared pathogen detection rates of culture and PCR. The main outcome of interest was a comparison of accuracy and time to final positive result. **Results:** In the total cohort, the pathogen detection rate in culture was 58.14% versus 34.88% in PCR (*p* = 0.03). Detection rates of bacteria in peritoneal dialysis patients were even higher by culture (70.83%) but comparably low by PCR (37.50%; *p* = 0.04). The majority of discordant results were in the Gram-positive spectrum (81.82%). Differential time to final positive result was 37.39 ± 16.75 h. **Conclusion:** Time gains by using PCR diagnostic have to be weighed against lower detection rates, mainly in Gram-positive infections.

## 1. Introduction

Peritonitis is one of the serious complications of peritoneal dialysis (PD). It shows a prevalence of 12–26% and is a major contributing cause of death in >15% of patients on PD [1,2]. Not only is PD-related (PDrP) peritonitis significantly associated with mortality [3], but it also leads to impaired ultrafiltration capacity and to conversion to long-term hemodialysis [4].

Prompt diagnosis and administration of appropriate antibiotics is the most important determinant for successful management in PDrP, which requires the identification of the causative pathogen.

Spontaneous bacterial peritonitis (SBP) is a typical infection of ascitic fluid in patients with cirrhosis that occurs in the absence of an obvious source of infection and is one of the most frequent predisposing factors for acute-on-chronic liver failure (ACLF) [5]. It is a life-threatening complication of cirrhosis with mortality rates ranging from 20 to 40% [6] and shows a highly variable clinical presentation. Current guidelines recommend prompt diagnostic testing for SBP by paracentesis in patients with cirrhosis and ascites presenting at a hospital [7].

Similar to PDrP, effective antibiotic therapy has to be started as soon as possible to reduce the frequency and severity of complications, because a delay in SBP diagnosis and treatment is associated with increased mortality [8]. However, given the broad spectrum of potential pathogens and increasing antibiotic resistance, empiric antibiotic treatment is challenging, and rapid identification of the causative microorganism is highly desirable.

Conventional microbiological culture is slow and fails to detect bacterial pathogens in a significant number of cases in patients with SBP and PDrP, which leads to a delay in diagnosis and a high number of false-negative test results. The Unyvero IAI application (Curetis GmbH, Holzgerlingen, Germany) is a multiplex PCR technology, which has been designed for the diagnosis of intra-abdominal infections (IAI) and delivers results within around five hours with automatic processing of the sample.

We here present a single-center comparison of the Unyvero IAI application to standard of care microbiological testing in patients with peritonitis undergoing peritoneal dialysis or on the background of liver cirrhosis. We address the question of whether the use of Unyvero IAI PCR in non-surgical peritonitis cases increases diagnostic accuracy and accelerates the time to positive result.

## 2. Patients and Methods

### 2.1. Study Population and Diagnostic Criteria

This retrospective study was performed at the University Hospital Bonn, Germany. Hospital patients with liver cirrhosis or patients undergoing peritoneal dialysis (i.e., continuous ambulatory peritoneal dialysis) with high suspicion of peritonitis, from whom specimen for detection of bacteria could be collected before the start of antibiotic treatment for peritonitis, were included. Specimen were collected between July 2017 and October 2020.

Microbiological diagnostic was carried out with culture as standard of care and with multiplex PCR application (Unyvero IAI).

A minority (*n* = 3) of patients received medical immunosuppression at the time of peritonitis due to the diagnosis underlying their renal disease (vasculitis (*n* = 2); graft failure (*n* = 1)). No patient had already received antibiotic treatment for peritonitis when specimen were collected.

Peritonitis was confirmed if

(a) polymorphonuclear leukocytes (PMN) count of ascitic fluid was ≥250 cells/µL in patients with liver cirrhosis in the absence of a contiguous source of infection or

(b) Two out of the three following criteria were met in patients on peritoneal dialysis:

– clinical features consistent with peritonitis (e.g., abdominal pain or cloudy dialysis effluent)
– dialysis effluent white cell count ≥100/µL (after a dwell time of at least 2 h) with ≥50% PMN
– a positive dialysis effluent bacterial culture [9].

If cell counts (measured by automated analyzer) were below this threshold in patients with liver cirrhosis, detection of a pathogen in the intra-abdominal fluid was considered peritonitis if the patient showed increased laboratory inflammatory parameters (C-reactive protein, leukocytes, neutrophils) and clinical signs of infection (one case).

Patients undergoing PD with concomitant cirrhosis were attributed to the PD-patient subgroup (one patient) because the presence of an indwelling peritoneal dialysis catheter excludes SBP by definition.

To take into account the different microbiological spectrum of bacteria in patients with liver cirrhosis and PDrP, we classified the microbiological spectrum of the cohort as follows:

Usually pathogenic: Enterobacterales, non-fermenters (Acinetobacter species), *Staphylococcus aureus*, Enterococcus species, Candida species.

Pathogenic only in PDrP and probable external contamination in liver cirrhosis: Corynebacteria species, Rothia species, *Staphylococcus epidermidis*, *Staphylococcus haemolyticus*, *Streptococcus mitis*.

Baseline patient characteristics of this study cohort were extracted from medical files and records.

### 2.2. Multiplex PCR Assay

The Unyvero IAI PCR cartridge offers a panel with a variety of bacterial and fungal pathogens typically responsible for intra-abdominal infections, along with common antibiotic resistance markers. The test was handled as recommended by the manufacturer. In brief, 180 μL of the collected liquid specimen (intra-abdominal aspirate taken under aseptic conditions) was added to a sample tube. After that, sample lysis was performed with a 30 min protocol, including mechanical, thermal, chemical, and enzymatic sample treatment. The lysed sample was further processed in the PCR-Cartridge (Unyvero intra-abdominal infection (IAI) multiplex PCR System, Curetis, Holzgerlingen, Germany). Internal controls were performed in every PCR run. Results were obtained after approximately 5 h and quantified by image processing with the manufacturer’s software (Unyvero operation software OS 3.0).

The pathogens and resistance markers that can be detected using the Unyvero IAI cartridge are shown in Supplementary Table S1.

### 2.3. Microbiological Cultures

For routine diagnostics intra-abdominal aspirates were collected under sterile conditions. Classically, the intra-abdominal aspirate was inoculated in PEDS medium blood culture flasks (Becton & Dickinson, Heidelberg, Germany) and incubated in a Bactec FX blood culture system (Becton & Dickinson, Heidelberg, Germany) for 5 days. Besides blood culture flasks, the native aspirate was plated on Columbia agar with 5% sheep blood, Mac Conkey agar, chocolate agar, and Sabouraud agar (all from Becton & Dickinson, Heidelberg, Germany), while 1 mL was pipetted into thioglycolate bouillon (Becton & Dickinson, Heidelberg, Germany). Additionally, Schaedler and kanamycin/vancomycin agar plates (Becton & Dickinson, Heidelberg, Germany) for anaerobic cultures were streaked with 0.5 mL of the liquid aspirate. Aerobic and anaerobic cultures were grown at 5% CO_2_ and 35 °C for at least 3 days.

The identification of pathogens was carried out using matrix-assisted laser desorption/ionization time-of-flight (MALDI-TOF) spectroscopy (bioMerieux, Nürtingen, Germany). Additionally, the antimicrobial susceptibility testing was performed with an automated antimicrobial susceptibility testing system, Vitek2 (bioMerieux). In the case of detection of anaerobe bacteria, susceptibility testing was carried out with a semiautomated microtiter broth dilution system (MICRONAUT; Merlin, Bornheim, Germany).

### 2.4. Outcome Assessment

Intraperitoneal aspirate for culture and PCR analysis were secured and sent simultaneously to the department of microbiology. Microbiological culture was defined as standard of care.

Time to final positive result was defined from the time of sample acquisition to the report of the test result.

We stratified results to concordant positive (culture and PCR positive) and discordant positive (culture positive and PCR negative or vice versa).

The main outcome of interest was a comparison of performance in terms of accuracy and time to final positive result.

### 2.5. Statistical Analysis

For characterization of the cohort, patients were assigned to subcohorts depending on whether peritonitis could be confirmed or not and then further characterized by their pre-existing conditions, including relevant comorbidities, results of blood analysis (with focus on parameters of inflammation, liver, and kidney function) and ascitic fluid analysis. Performance analysis of ascitic fluid culture and PCR was based on pathogen detection rate, which was calculated by dividing the number of cases with the detection of one (or more) causative pathogen(s) (=true positive) by the number of cases with peritonitis evaluated with the respective modality (=true positive + false positive). The pathogens detected by ascitic fluid culture and PCR are presented for the total cohort and the subcohorts of peritoneal dialysis and cirrhosis separately. Categorical variables (i.e., pre-existing conditions, including comorbidities, microbiological spectrum of culture, and PCR) are presented as frequency and absolute numbers in parentheses, while continuous data (i.e., laboratory values, time to final positive result) are given as mean ± standard deviation. Statistical analysis of categorial variables was performed using Pearson’s Chi-squared test. Statistical comparison of continuous data (i.e., time to final positive result) was carried out by Student’s *t* test. A two-tailed *p*-value of 0.05 was considered statistically significant. SPSS version 29 was used for statistical analyses.

## 3. Results

### 3.1. Characteristics of the Cohort and Performance of Culture and PCR

We included 47 patients with clinical high suspicion of peritonitis in our study. Of those, 43 patients finally met the diagnostic criteria of peritonitis. The cohort was well balanced in terms of the underlying disease (55.32% PD patients, 46.81% patients with cirrhosis). General information, as well as laboratory results of the cohort, are given in Table 1.

In the total cohort, culture detected 31 pathogens in 28 patients and PCR 17 pathogens in 15 patients. A total of 87.50% of these detected pathogens were classified as causative. Pathogen detection rates in the total cohort varied significantly. Culture identified causative pathogens in 58.14% and PCR in 34.88% (*p* = 0.03). This difference was of statistical significance in PD patients as well (culture: 70.83%; PCR 37.50%; *p* = 0.02), whereas the difference was less pronounced in patients with liver cirrhosis (culture: 42.11%; PCR: 31.58%; *p* = 0.50). Only in one case was Vancomycin resistance detected by PCR which was later confirmed by culture results in enterococcal peritonitis in a patient with cirrhosis.

The rate of major complications during hospital admission was comparable between concordant positive and only culture-positive specimen for in-house mortality (two specimens concordant positive, one specimen only culture positive) and switch to hemodialysis (two patients, both concordant positive).

Time to final positive result (for causative pathogens only) was longer in culture compared to PCR (59.02 ± 13.78 h vs. 19.06 ± 9.85 h; *p* < 0.001), Table 2.

**Figure 1:**
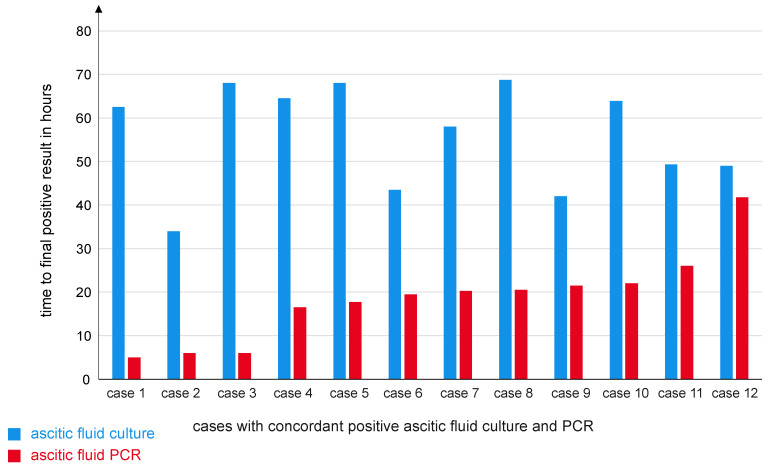
Time to positive result of culture and PCR.

To better compare this time difference, all concordantly positive test results in culture and PCR with available data concerning time to final positive result for both were included in a performance analysis (*n* = 12). Differential time to final positive result was 37.39 ± 16.75 h (Figure 1).

### 3.2. Microbiological Spectrum of the Cohort

Pathogens detected by culture were mainly Gram-positive (64.52%), whereas pathogens detected by PCR were Gram-positive and Gram-negative with comparable proportions (52.94% and 47.06%, respectively). Of Gram-negative bacteria, the majority was identified as Enterobacterales in culture (22.58%) and PCR testing (35.29%), respectively. Gram-positive bacteria were mainly identified as CNS and Enterococcus species by both, culture (29.03% and 16.13%, respectively) and PCR (17.65% and 29.41%, respectively). Table 3 gives a detailed description of the microbiological spectrum of the cohort.

In patients with PDrP, we detected 17 pathogens by culture. Culture revealed 64.71% Gram-positive and 35.29% Gram-negative bacteria. CNS were the predominant Gram-positive bacteria (35.29%). Among the Gram-negative bacteria, Enterobacterales and Acinetobacter species were identified as the causative pathogens with equal proportions of 17.65%. By contrast, PCR detected only 10 pathogens, most of them Gram-negative (60%) and only 40% Gram-positive. For CNS, culture identified six cases and PCR two cases. Enterococci could be detected by PCR, whereas their species could not be further delineated (Table 4). In one case of PDrP, culture detected *Enterococcus faecium*, while PCR additionally detected *Escherichia coli*. This case was considered as polymicrobial peritonitis.

In patients with liver cirrhosis (non-PD), 14 pathogens were identified by culture. Among them, 28.57% of pathogens were Gram-negative and 64.29% Gram-positive bacteria, with one case (7.14%) of candida infection. PCR detected seven pathogens among patients with liver cirrhosis (non-PD). A total of 57.14% of these pathogens were Gram-positive bacteria (predominantly Enterococci). Similar to PDrP, in patients with liver cirrhosis, Enterococcus species was detected by PCR but could not be attributed to a specific species (Table 5). All Gram-negative bacteria detected by PCR were Enterobacterales. In two cases, both culture and PCR detected Enterococcus species and CNS. Detection of CNS was suspected to be due to contamination in these cases. In a third case with polymicrobial test results, culture detected Enterococcus species and Candida species, while PCR missed out Candida. The latter case was regarded as true polymicrobial peritonitis.

### 3.3. Concordance of Test Results

We identified discordantly positive results (culture positive and PCR negative findings) in 16 cases. Those discordant results were predominantly found in Gram-positive (81.25%) and less frequent in Gram-negative spectrum (18.18%). In twelve of these sixteen cases with positive culture and negative PCR, pathogens (i.e., cases with Enterobacter species, CNS, *Staphylococcus aureus*, Streptococcus species) were missed although the pathogen was included in the Unyvero IAI panel (Table 6). For the other four cases, PCR did not have the chance to detect the pathogens a priori, as the respective pathogens were out of the PCR panel. In one case, PCR detected CNS, whereas the culture remained negative. In one case of polymicrobial PDrP, PCR additionally detected *Escherichia coli*, which was missed out by culture. Culture and PCR were concordantly positive in 15 cases and concordantly negative in 18 cases.

## 4. Discussion

We describe the performance of a multiplex PCR Application (Unyvero IAI) in routine diagnostic setting in a cohort of patients with peritonitis. We compared it to microbiological culture as standard of care.

Multiplex molecular panels have the possibility to identify pathogens and antimicrobial resistance within a significantly shorter amount of time than microbiological culture. They have been tested in several infectious diseases and biomaterials such as gastrointestinal infections (feces) [10], bloodstream infections [11], infections of the central nervous system (cerebrospinal fluid) [12], periprosthetic joint infection [13], and respiratory tract infections [14]. We here analyzed ascitic fluid in a clearly defined cohort of patients with non-surgical peritonitis on the background of liver cirrhosis and peritoneal dialysis. This is particularly relevant given that the bacterial spectrum between entities differs.

So far, reports of Unyvero PCR testing of intra-abdominal infections are limited to two studies [15,16]. Both studies showed that the Unyvero IAI PCR was able to detect additional microorganisms compared to culture in Gram-positive and Gram-negative spectrum. Both studies included very heterogeneous clinical samples like peritoneal fluid, bile, pus, but also swabs in patients with peritonitis originating from various intra-abdominal infections (e.g., intra-abdominal abscesses, necrotizing pancreatitis, and infected pancreatic or biliary fistulas).

Typically, SBP is monobacterial caused by mostly enteric Gram-negative bacteria, such as *Escherichia coli*, followed by *Klebsiella pneumoniae*, *Staphylococcus aureus*, *Enterococcus faecalis*, and *Enterococcus faecium* [17,18]. Particularly in nosocomial SBP, there has been a shift towards Gram-positive bacteria and multidrug-resistant organisms leading to an attenuated response to initial antibiotic treatment [5,19,20,21].

Gram-positive and Gram-negative organisms can both cause PD-related peritonitis with Coagulase-negative staphylococci and *Staphylococcus aureus* being the most frequent causative organisms due to translocation from the skin along the dialysis catheter or contact contamination. In nosocomial infections, vancomycin-resistant Enterococcus (VRE), methicillin-resistant *Staphylococcus aureus*, and fungal organisms should be considered [22,23].

We could demonstrate a significant statistical difference in pathogen detection rate between culture and PCR in PD patients, whereas in patients with liver cirrhosis, detection rates were comparable. Within our study, among the pathogens included in the Unyvero IAI PCR panel, mainly CNS could not be identified. This might be attributable to a thicker peptidoglycan layer of Gram-positive bacteria and thus an ineffective lysis process by the application [24]. In addition, the threshold for CNS may be set quite high, which may lead to a loss in sensitivity. Of course, lowering the threshold for CNS detection by PCR could result in an increase in false-positive findings resulting from contamination.

Previous studies show very different results regarding the accuracy of the PCR application. This seems to depend largely on characteristics of the cohort and biomaterial, as well as the expected microbial spectrum. In an analysis of bronchoalveolar lavage, tracheal aspirates and pleural fluids from neonates and children, Unyvero PCR yielded a sensitivity of 73.1% and a specificity of 97.9% compared to culture method. Interestingly, sensitivity and specificity for Gram-positive bacteria was worse [24]. However, using the same Unyvero PCR in a cohort of adults with suspected pneumonia, another study calculated very good sensitivity (95.7%) and very low specificity (32.6%) [25]. In a similar cohort, a newer generation of Unyvero cartridge provided a better sensitivity and specificity (88.8% sensitivity and 94.9% specificity) [26].

One multicenter, randomized controlled trial revealed that multiplex bacterial PCR examination of bronchoalveolar lavage decreased the duration of inappropriate antibiotic therapy of patients admitted to hospital with pneumonia, who were at risk of Gram-negative bacterial infection [27].

In contrast to the results with respiratory focus, evidence in other infections is much clearer. Several different studies showed a non-superiority of the multiplex PCR over standard methods in implant and tissue infection [13,28,29].

One of the challenges arising from these previous studies is the lack of generalizability, since PCR diagnostics showed varying accuracy depending on the disease entity. Interdisciplinary diagnostic stewardship and more clinical trials are important to providing PCR testing for those patients that will benefit from its use. Apart from diagnostic accuracy, the duration of the diagnostic process also has to be considered. Within our study, time to final positive result was much longer in culture compared to PCR, which might delay guided therapeutic decisions to a specific antibiotic therapy. Of note, because Unyvero PCR was performed only during normal office hours, there was a delay in testing if the specimen were collected outside normal office hours, which, however, might reflect real world practice.

Although microbiological diagnostic should be performed before initiation of treatment, this might sometimes be challenging in clinical routine, especially for analysis of intraperitoneal fluid. In this context, PCR might also render positive results after the start of antibiotic treatment, as suggested by one study of patients with prosthetic joint infection, which showed a benefit of detecting pathogens by PCR in patients who already received antibiotic therapy [30].

In conclusion, our study provides insights into the performance of Unyvero IAI PCR application compared to culture in a well-defined cohort of peritonitis patients. Our data are in line with previous evidence, which shows a very variable performance in terms of diagnostic accuracy and appears to be largely dependent on the type of infection, as well as the analyzed biomaterial. Due to the limited performance of PCR in the detection of Gram-positive bacteria, especially CNS, we could not derive clinical benefit of the system in diagnosing Gram-positive pathogens in peritoneal dialysis patients.

In up to 50% of SBP, both blood and ascites cultures remain negative [31]. New molecular methods, such as Unyvero PCR, might become a helpful diagnostic tool for the improvement of microbial sensitivity in patients with SBP in the context of liver cirrhosis. However, our results do not support that Unyvero IAI PCR enhances the sensitivity of microbiological testing. Still, the advantage by gaining time may be significant, because earlier treatment improves outcomes of SBP [6]. Although our results cannot be generalized, our data advocate for an interdisciplinary approach of clinicians and microbiologists to ensure the best and fastest diagnostic for each patient.

For patients with PDrP, the potential additive value of Unyvero IAI PCR is a faster diagnostic of Gram-negative bacteria with a substantial risk in missing Gram-positive pathogens.

Based on our results, Unyvero PCR may improve diagnosis in peritonitis patients, especially facilitating early specific antibiotic treatment, and therefore might be a useful adjunct tool. However, a negative test result in SBP and PDrP needs to be verified by conventional bacterial culture. Our data do not support the sole use of Unyvero PCR in patients with suspected peritonitis.

## Figures and Tables

**Table 1:** Characteristics of the cohort.

	No Peritonitis (*n* = 4)	Peritonitis (*n* = 43)	All (*n* = 47)
General information			
Pre-existing conditions			
PD	50% (2/4)	55.81% (24/43)	55.32% (26/47)
cirrhosis	75% (3/4)	44.19% (19/43)	46.81% (22/47)
Cause of free fluid			
PD ^1^	50% (2/4)	55.81% (24/43)	55.32% (26/47)
cirrhosis ^1^	50% (2/4)	44.19% (19/43)	44.68% (21/47)
Clinical details of cirrhosis patients			
prior SBP	33.33% (1/3)	15.79% (3/19)	18.18% (4/22)
esophageal varices	100% (3/3)	64.29% (9/14)	70.59% (12/17)
prior TIPS	33.33% (1/3)	11.11% (2/18)	14.29% (3/21)
HCC	0% (0/3)	33.33% (5/15)	27.78% (5/18)
MELD	29 ± 14.14	23.12 ± 8.01	23.74 ± 8.46
Cause of CKD in PD patients			
Alport’s syndrome	0% (0/2)	8.33% (2/24)	7.69% (2/26)
polycystic kidney disease	50% (1/2)	12.50% (3/24)	15.38% (4/26)
ANCA-associated vasculitis	0% (0/2)	20.83% (5/24)	19.23% (5/26)
diabetic nephropathy	0% (0/2)	4.17% (1/24)	3.85% (1/26)
hypertensive nephrosclerosis	0% (0/2)	12.50% (3/24)	11.54% (3/26)
others	0% (0/2)	25% (6/24)	23.08% (6/26)
unknown	50% (1/2)	16.67% (4/24)	19.23% (5/26)
Further clinical details of PD patients			
duration of PD [months]	1.50 ± 0.71	27.86 ± 34	25.57 ± 33.30
definite discontinuation of PD due to peritonitis	not applicable	8.33% (2/24)	[8.33% (2/24)]
Blood analysis			
sodium [mmol/L]	131.50 ± 3.54	134.19 ± 5.88	134 ± 5.75
creatinine [mg/dL]	2.30 ± 2.26	3.82 ± 2.60	3.70 ± 2.57
albumin [g/L]	25 ± 2.83	27.12 ± 5.98	26.88 ± 5.70
bilirubin [mg/dL]	12.65 ± 7.57	2.80 ± 3.02	3.79 ± 4.51
GOT [U/L]	119.50 ± 40.31	175 ± 442.13	168.83 ± 415.81
GPT [U/L]	81 ± 63.64	56 ± 54.35	58.63 ± 53.97
gGT	110.50 ± 105.36	187.63 ± 159.16	179.06 ± 153.71
alkaline phosphatase [U/L]	279.50 ± 226.98	236.91 ± 172.14	243.46 ± 171.01
INR	1.80 ± 0.61	1.31 ± 0.62	1.35 ± 0.63
CRP [mg/L]	20 ± 16.64	84.36 ± 95.20	79.77 ± 93.24
leucocytes [G/L]	16.60 ± 11.80	11.38 ± 7.92	11.75 ± 8.17
thrombocytes [G/L]	239 ± 130.68	208.87 ± 128.42	211.07 ± 127.17
Analysis of free intra-abdominal fluid			
leucocytes [per µL]	382.25 ± 381.67	6204.02 ± 8550.76	5708.55 ± 8334.48
absolute PMN count [per µL]	148.75 ± 148.86	5116.35 ± 7489.05	4693.57 ± 7292.02
relative PMN count	0.41 ± 0.10	0.76 ± 0.16	0.73 ± 0.18

PD: peritoneal dialysis, SBP: spontaneous bacterial peritonitis, TIPS: transjugular intrahepatic portosystemic shunt, MELD: model of end-stage liver disease, HCC: hepatocellular carcinoma, INR: international normalized ratio, CRP: C-reactive protein, PMN: polymorphonuclear cells. CKD: chronic kidney disease, ANCA: Anti-neutrophil cytoplasmic antibodies, GOT glutamic oxaloacetic transaminase: GPT, gGT: Gamma-glutamyltransferase. ^1^ One patient with liver cirrhosis undergoing renal replacement therapy by PD was assigned to the PD group.

**Table 2:** Performance of culture and PCR.

	Culture	PCR	*p* ^1^
Pathogen detection rate			
total cohort	58.14% (25/43)	34.88% (15/43)	0.03
PD-patients	70.83% (17/24)	37.50% (9/24)	0.02
cirrhosis patients (non-PD)	42.11% (8/19)	31.58% (6/19)	0.50
	Culture	PCR	*p* ^2^
time to final positive result [h]	59.02 ± 13.78	19.06 ± 9.85	<0.001

PD: peritoneal dialysis, ^1^ statistical analysis by Pearson’s Chi-squared test, ^2^ statistical analyses based by Student’s *t* test.

**Table 3:** Microbiological spectrum of total cohort.

	Culture ^1^	PCR ^2^
Gram-negative bacteria	32.26% (10/31)	52.94% (9/17)
Enterobacteriaceae	22.58% (7/31)	35.29% (6/17)
*Escherichia coli*	9.68% (3/31)	23.53% (4/17)
Klebsiella species	3.23% (1/31)	5.88% (1/17)
Enterobacter species	6.45% (2/31)	5.88% (1/17)
Citrobacter species ^3^	3.23% (1/31)	0% (0/17)
Acinetobacter species	9.68% (3/31)	17.65% (3/17)
Gram-positive bacteria	64.52% (20/31)	47.06% (8/17)
Corynebacterium species ^3^	3.23% (1/31)	0% (0/17)
*Staphylococcus aureus*	3.23% (1/31)	0% (0/17)
CNS	29.03% (9/31)	17.65% (3/17)
Streptococcus species	6.45% (2/31)	0% (0/17)
Enterococcus species	16.13% (5/31)	29.41% (5/17)
*Enterococcus faecalis*	6.45% (2/31)	0% (0/17)
*Enterococcus faecium*	6.45% (2/31)	0% (0/17)
not specified	3.23% (1/31)	29.41% (5/17)
Rothia species ^3^	6.45% (2/31)	0% (0/17)
Candida species	3.23% (1/31)	0% (0/17)

CNS: coagulase-negative Staphylococcus species, ^1^ detection of two pathogens in three cases, ^2^ detection of two pathogens in two cases, ^3^ out of Unyvero IAI-PCR-Panel.

**Table 4:** Microbiological spectrum of PD patients.

	Culture	PCR ^1^
Gram-negative bacteria	35.29% (6/17)	60% (6/10)
Enterobacteriaceae	17.65% (3/17)	30% (3/10)
*Escherichia coli*	5.88% (1/17)	20% (2/10)
Klebsiella species	0% (0/17)	0% (0/10)
Enterobacter species	5.88% (1/17)	10% (1/10)
Citrobacter species ^2^	5.88% (1/17)	0% (0/10)
Acinetobacter species	17.65% (3/17)	30% (3/10)
Gram-positive bacteria	64.71% (11/17)	40% (4/10)
Corynebacterium species ^2^	5.88% (1/17)	0% (0/10)
*Staphylococcus aureus*	0% (0/17)	0% (0/10)
CNS	35.29% (6/17)	20% (2/10)
Streptococcus species	5.88% (1/17)	0% (0/10)
Enterococcus species	11.76% (2/17)	20% (2/10)
*Enterococcus faecalis*	5.88% (1/17)	0% (0/10)
*Enterococcus faecium*	5.88% (1/17)	0% (0/10)
not specified	0% (0/17)	20% (2/10)
Rothia species ^2^	5.88% (1/17)	0% (0/10)
Candida species	0% (0/17)	0% (0/10)

CNS: coagulase-negative Staphylococcus species, ^1^ detection of two pathogens in one case, ^2^ out of Unyvero-IAI PCR-Panel.

**Table 5:** Microbiological spectrum of patients with liver cirrhosis (non-PD).

	Culture ^1^	PCR ^2^
Gram-negative bacteria	28.57% (4/14)	42.86% (3/7)
Enterobacteriaceae	28.57% (4/14)	42.86% (3/7)
*Escherichia coli*	14.29% (2/14)	28.57% (2/7)
Klebsiella species	7.14% (1/14)	14.29% (1/7)
Enterobacter species	7.14% (1/14)	0% (0/7)
Citrobacter species ^3^	0% (0/14)	0% (0/7)
Acinetobacter species	0% (0/14)	0% (0/7)
Gram-positive bacteria	64.29% (9/14)	57.14% (4/7)
Corynebacterium species ^3^	0% (0/14)	0% (0/7)
*Staphylococcus aureus*	7.14% (1/14)	0% (0/7)
CNS	21.43% (3/14)	14.29% (1/7)
Streptococcus species	7.14% (1/14)	0% (0/7)
Enterococcus species	21.43% (3/14)	42.86% (3/7)
*Enterococcus faecalis*	7.14% (1/14)	0% (0/7)
*Enterococcus faecium*	7.14% (1/14)	0% (0/7)
not specified	7.14% (1/14)	42.86% (3/7)
Rothia species ^3^	7.14% (1/14)	0% (0/7)
Candida species	7.14% (1/14)	0% (0/7)

CNS: coagulase-negative Staphylococcus species, ^1^ detection of two pathogens in three cases, ^2^ detection of two pathogens in one case, ^3^ out of Unyvero-IAI PCR-Panel.

**Table 6:** Discordant positive cases: culture positive/PCR negative (*n* = 16).

	Culture
Gram-negative bacteria	12.50% (2/16)
Enterobacteriaceae	12.50% (2/16)
*Escherichia coli* ^1^	0% (0/16)
Klebsiella species	0% (0/16)
Enterobacter species ^1^	6.25% (1/16)
Citrobacter species ^2^	6.25% (1/16)
Acinetobacter species	0% (0/16)
Gram-positive bacteria	81.25% (13/16)
Corynebacterium species ^2^	6.25% (1/16)
*Staphylococcus aureus* ^1^	6.25% (1/16)
CNS ^1^	43.75% (7/16)
Streptococcus species ^1^	12.50% (2/16)
Enterococcus species^1^	0% (0/16)
*Enterococcus faecalis*	0% (0/16)
*Enterococcus faecium*	0% (0/16)
not specified	0% (0/16)
Rothia species ^2^	12.50% (2/16)
Candida species ^1^	6.25% (1/16)

CNS: coagulase-negative Staphylococcus species, ^1^ within Unyvero-PCR-Panel, ^2^ out of Unyvero-PCR-Panel.

## Data Availability

The datasets generated during and/or analyzed during the current study are available from the corresponding author on reasonable request.

## Supplementary Materials

The following supporting information can be downloaded at: https://www.mdpi.com/article/10.3390/jcm14082641/s1, Table S1: Full list of pathogens and resistance genes.
