# Supplementary material for: Multiplex PCR for Microbiological Testing in Patients with Peritoneal Dialysis- and Liver Cirrhosis-Related Peritonitis: Faster, but Less Sensitive
Source: J Clin Med. 2025 Apr 11;14(8):2641. doi: 10.3390/jcm14082641 (PMC12028186; doi:10.3390/jcm14082641)
Supplement: Supplementary file 1 [file jcm-14-02641-s001.zip › jcm-3551651-supplementary.pdf]

Supplemental Table 1: Full list of pathogens and resistance genes

**Spectrum of Unyvero IAI PCR**
**Enterobacterales**<br>E. coli<br>Klebsiella aerogenes<br>Enterobacter cloacae complex<br>Klebsiella pneumoniae<br>Klebsiella oxytoca<br>Klebsiella varicola<br>Proteus species
**Anaerobic/facultative anaerobic bacteria**<br>Aeromonas spp.<br>Bacteroides fragilis group<br>Bacteroides species / Prevotella species<br>Clostridiodes difficile<br>Clostridium perfringens<br>Finegoldia magna<br>Cutibacterium acnes
**Gram-positive bacteria**<br>Staphylococcus aureus<br>coagulase-negative staphylococci<br>Streptococcus species<br>Enterococcus species

Enterococcus faecalis
**Non-fermenting bacteria**<br><br>Acinetobacter baumanii complex<br><br>Pseudomonas aeruginosa
**Fungi**<br><br>Candida species<br><br>Candida albicans<br><br>Candida glabrata<br><br>Candida tropicalis<br><br>Candida krusei
**Resistance (Gene)**<br><br>aacA4, ctx-M, fosA3, imp, kpc, mcr-1, ndm, nimA, nimB, mecA, mecC, oxa-23, oxa-24/40, oxa-48, oxa-58, qnrA, qnrB, qnrS, stx1/2, tcdB, tetA, vanA, vanB, vim, tcdB.
